# Interleukin-6, Proteinuria, and Arterial Stiffness in Preeclampsia: Distinct Inflammatory, Renal, and Vascular Associations

**DOI:** 10.3390/biomedicines14092069

**Published:** 2026-09-15

**Authors:** Izabella Petre, Mircea Iurciuc, Florina Buleu, Daian Popa, Luciana Marc, Tiberiu Buleu, Ioana Marin, Ramona Dragomir, Narcisa Carmen Mladin, Marina Adriana Mercioni, Ion Petre

**Affiliations:** 1 Department of Obstetrics and Gynecology, “Victor Babes” University of Medicine and Pharmacy, 2 Eftimie Murgu Square, 300041 Timisoara, Romania; 2 Department of Clinic Nursing, “Victor Babes” University of Medicine and Pharmacy, Eftimie Murgu Square 28 2, 300041 Timisoara, Romania; 3 Cardiology Department, “Victor Babes” University of Medicine and Pharmacy, 300041 Timisoara, Romania; 4 Research Center for Medical Communication, “Victor Babes” University of Medicine and Pharmacy of Timisoara, 300041 Timisoara, Romania; 5 Department of Internal Medicine II, Division of Nephrology, “Victor Babes” University of Medicine and Pharmacy, 300041 Timisoara, Romania; 6 Faculty of General Medicine, “Victor Babes” University of Medicine and Pharmacy, 300041 Timisoara, Romaniamarina.mercioni@student.umft.ro (M.A.M.); 7 Department of Microbiology, “Victor Babes” University of Medicine and Pharmacy, Eftimie Murgu Sq. No. 30 2, 300041 Timisoara, Romania; ioana.marin@umft.ro; 8 “Alessandrescu-Rusescu” National Institute for Mother and Child Health, bd. Lacul Tei nr.120, 020395 Bucharest, Romania; 9 Department of General Medicine, “Vasile Goldis” Western University, B-dul Revoluției Nr. 96, 310025 Arad, Romania; mladin.narcisa@uvvg.ro; 10 Applied Electronics Department, Faculty of Electronics, Telecommunications and Information Technologies, 22 Politehnica University Timișoara, 300223 Timisoara, Romania; 11 Department of Functional Sciences, Medical Informatics and Biostatistics Discipline, “Victor Babes” University of Medicine and Pharmacy, Eftimie Murgu Square 2, 300041 Timisoara, Romania

**Keywords:** preeclampsia, interleukin-6, IL-6, arterial stiffness, pulse wave velocity, augmentation index, proteinuria, maternal cardiovascular function, pregnancy, inflammation

## Abstract

**Background**: Preeclampsia is characterized by systemic inflammation, endothelial dysfunction, and progressive abnormalities in the maternal vasculature. Interleukin-6 (IL-6) may indicate inflammatory activity, while aortic pulse wave velocity (PWVao) and augmentation index (AIx) offer complementary assessments of arterial stiffness and vascular function. This study examined the relationships among IL-6, proteinuria, and arterial stiffness in women with preeclampsia. **Methods**: This observational study involved 87 pregnant women with preeclampsia who were evaluated at the Emergency Clinical Hospital “Pius Brînzeu” in Timișoara, Romania. Arterial stiffness was measured non-invasively with the Arteriograph system during the second trimester (20–24 weeks) and the third trimester (32–36 weeks). PWVao served as the primary marker of arterial stiffness, whereas AIx provided a complementary measure of arterial wave reflection. Serum IL-6 levels were quantified by enzyme-linked immunosorbent assay, and 24 h proteinuria was recorded. Associations among inflammatory, renal, and vascular parameters were examined using Spearman correlation analyses. Multivariable linear regression then identified independent predictors of third-trimester PWVao. **Results**: The mothers had a mean age of 37.59 ± 2.65 years, and the mean BMI was 30.41 ± 2.70 kg/m^2^. PWVao rose significantly between the second and third trimesters, from 10.94 ± 1.36 m/s to 12.46 ± 1.70 m/s (*p* < 0.001). AIx also increased, from 1.26 ± 0.85% to 3.66 ± 1.32% (*p* < 0.001). The mean serum IL-6 concentration was 31.01 ± 35.61 pg/mL. No significant correlation was found between IL-6 and PWVao in either the second trimester (ρ = −0.100, *p* = 0.356) or the third trimester (ρ = −0.100, *p* = 0.358), and IL-6 was not significantly correlated with AIx. A significant positive correlation was observed between IL-6 and 24 h proteinuria (ρ = 0.337, *p* = 0.0014). Proteinuria itself showed no significant association with PWVao or AIx. In the multivariable analysis, only BMI independently predicted third-trimester PWVao (β = 0.634, 95% CI: 0.469–0.799, *p* < 0.001). Age, systolic blood pressure, proteinuria, and IL-6 showed no independent association with PWVao. Nor were IL-6, proteinuria, or third-trimester PWVao significantly associated with birth weight or the 1 min Apgar score. **Conclusions**: Among women with preeclampsia, arterial stiffness increased progressively throughout pregnancy. Systemic inflammation, measured by IL-6, was more strongly associated with renal involvement than with mechanical arterial stiffness. BMI was the main independent determinant of third-trimester PWVao. Together, these results support a multidimensional view of preeclampsia, in which inflammatory, renal, metabolic, and vascular abnormalities form related yet partly distinct pathways. Assessing IL-6, proteinuria, BMI, and arterial stiffness together may improve maternal cardiovascular phenotyping and help establish future risk-stratification strategies.

## 1. Introduction

Preeclampsia (PE) is a multisystemic hypertensive disorder that occurs exclusively during pregnancy and is one of the leading causes of maternal and perinatal morbidity and mortality worldwide [1]. With an incidence rate of approximately 2–8% of all pregnancies, it contributes significantly to preterm birth, fetal growth restriction, placental abruption, maternal organ failure, and long-term cardiovascular conditions [1,2,3]. Despite remarkable advances in obstetric care, the exact pathogenic mechanisms underlying the development and progression of preeclampsia remain unknown. There is a growing body of evidence supporting the view that preeclampsia should be considered a syndrome characterized not only by hypertension but also by generalized endothelial dysfunction, systemic inflammation, oxidative stress, and vascular damage [4].

Among the inflammatory mediators linked to the pathogenesis of preeclampsia, interleukin-6 (IL-6) has attracted considerable interest. Activated monocytes, macrophages, endothelial cells, vascular smooth muscle cells, adipocytes, and placental trophoblasts all produce this pleiotropic cytokine. In normal physiological conditions, IL-6 helps regulate immune responses and supports tissue repair. When produced excessively, though, it can drive endothelial activation, oxidative stress, leukocyte recruitment, and vascular remodeling [5]. Studies have consistently found higher maternal IL-6 concentrations in women with preeclampsia, associating them with disease severity, endothelial injury, and adverse maternal outcomes [6,7]. These findings suggest that IL-6 may function as a biomarker of systemic inflammation and vascular dysfunction during pregnancy.

During a normal pregnancy, the maternal cardiovascular system changes substantially: systemic vascular resistance falls, arterial compliance rises, and central hemodynamics shift to maintain adequate uteroplacental perfusion [8]. In pregnancies complicated by hypertension, these adjustments are impaired. Arterial stiffness increases, central blood pressure rises, and vascular compliance declines. These changes may appear before the clinical signs of hypertensive disorders, making them promising indicators of early maternal cardiovascular dysfunction [9].

Arterial stiffness is now recognized as a useful non-invasive indicator of vascular health and cardiovascular risk [10,11,12]. Carotid–femoral pulse wave velocity (PWV) remains the reference standard for assessing it, while the augmentation index (AIx) adds information about arterial wave reflection and peripheral vascular function. Central systolic blood pressure (cSBP) and central pulse pressure (cPP) offer another view of the hemodynamic load placed on the cardiovascular system and may capture vascular dysfunction more accurately than conventional peripheral blood pressure measurements [13,14]. Evidence is accumulating that women who later develop hypertensive disorders of pregnancy have greater arterial stiffness during the second and third trimesters. This supports the hypothesis that vascular dysfunction occurs before the disease becomes clinically apparent [15].

Although inflammation and endothelial dysfunction are recognized as fundamental mechanisms in the development of preeclampsia, only a limited number of studies have investigated the interplay between circulating inflammatory biomarkers, particularly IL-6, arterial stiffness, central hemodynamics, and adverse pregnancy outcomes within the same study population [16,17]. A better understanding of the interplay between systemic inflammation and maternal vascular dysfunction may provide important insights into the pathophysiology of hypertensive disorders of pregnancy and improve early risk stratification for adverse maternal and neonatal outcomes.

Therefore, this study investigated central hemodynamic parameters, arterial stiffness, and serum IL-6 concentrations in women with preeclampsia. It also examined whether IL-6, pulse wave velocity, augmentation index, and central blood pressure were associated with maternal clinical characteristics and adverse pregnancy outcomes, with the aim of exploring their potential relationships with maternal cardiovascular dysfunction and risk stratification during pregnancy.

## 2. Materials and Methods

### 2.1. Study Design and Population

This cross-sectional observational study was conducted at the Departments of Obstetrics and Gynecology of the Emergency Clinical Hospital “Pius Brînzeu”, Timișoara, Romania, in collaboration with the Victor Babeș University of Medicine and Pharmacy, Timișoara, Romania. The study included 87 pregnant women diagnosed with preeclampsia and was designed to investigate the association between systemic inflammation, assessed by serum interleukin-6 (IL-6) concentrations, and arterial stiffness in women with preeclampsia.

Preeclampsia was diagnosed according to the recommendations of the International Society for the Study of Hypertension in Pregnancy (ISSHP) [18] and the American College of Obstetricians and Gynecologists (ACOG) [19]. The diagnosis was based on the development of new-onset hypertension after 20 weeks of gestation, defined as a systolic blood pressure ≥ 140 mmHg and/or a diastolic blood pressure ≥ 90 mmHg measured on two separate occasions at least four hours apart, accompanied by proteinuria (≥300 mg/24 h) or evidence of maternal organ dysfunction according to current international diagnostic criteria.

**Figure 1:**
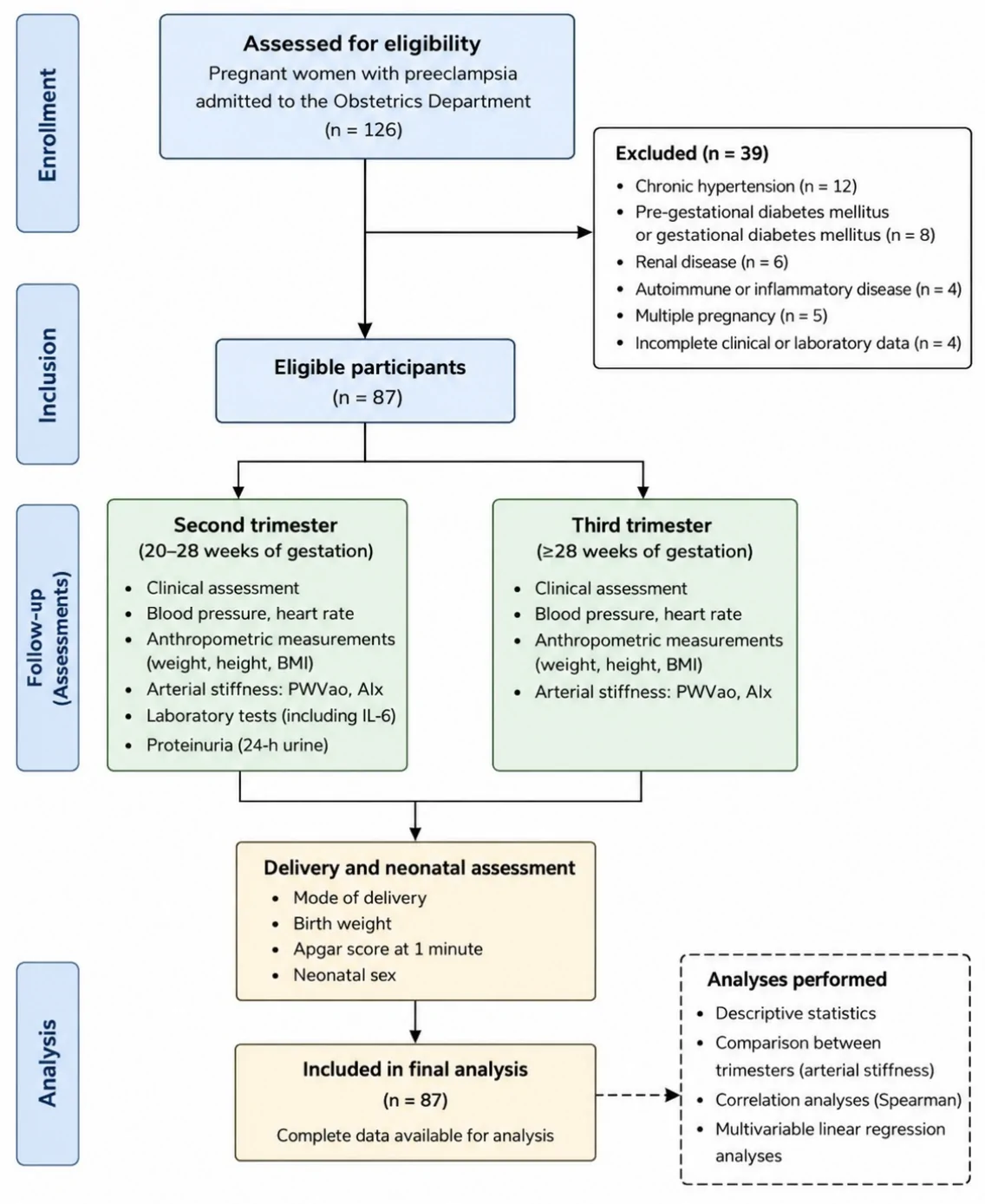
Study flowchart illustrating participant selection, clinical assessments, and analytical workflow.

The study workflow, including participant eligibility, inclusion and exclusion process, longitudinal vascular assessments performed during the second and third trimesters, neonatal outcome evaluation, and statistical analyses, is illustrated in Figure 1.

The study protocol was approved by the Ethics Committee of the Emergency Clinical Hospital “Pius Brînzeu”, Timișoara, Romania (Decision No. 54/20 April 2017). Written informed consent was obtained from all participants before enrollment, and the study was conducted in accordance with the ethical principles of the Declaration of Helsinki.

### 2.2. Eligibility Criteria

Eligible participants were women with a singleton pregnancy, a confirmed diagnosis of preeclampsia, a gestational age of at least 20 weeks, complete clinical, laboratory, and hemodynamic assessments, and available serum IL-6 measurements together with arterial stiffness evaluations.

Women were excluded if they had pre-existing chronic hypertension, chronic kidney disease, autoimmune disorders, acute infectious diseases, multiple pregnancy, major fetal congenital anomalies, pre-existing diabetes mellitus or gestational diabetes mellitus or incomplete clinical or laboratory records.

### 2.3. Clinical Assessment

We prospectively recorded demographic, anthropometric, obstetric, and clinical characteristics using standardized case-report forms and verified them against electronic medical records. Collected demographic and anthropometric variables included maternal age, height, body weight, and body mass index (BMI), while clinical assessment comprised smoking status, systolic blood pressure, diastolic blood pressure, and heart rate.

Blood pressure measurements were obtained according to current clinical recommendations after an adequate resting period using appropriately sized cuffs. Obstetric and neonatal outcomes included the mode of delivery, neonatal sex, birth weight, and the Apgar score at one minute after birth.

### 2.4. Hemodynamic Assessment

Arterial stiffness and central hemodynamic parameters were assessed non-invasively using the Arteriograph (v3.0.0.3; TensioMed Ltd., Budapest, Hungary), a validated oscillometric device that estimates vascular function by analyzing oscillometric pulse waves recorded from the upper arm. The system measures aortic pulse wave velocity (PWVao), brachial augmentation index (AIx), central systolic blood pressure (cSBP), central pulse pressure (cPP), and additional indices of arterial function.

Hemodynamic assessments were performed during the second trimester (20–24 weeks of gestation) and repeated during the third trimester (32–36 weeks of gestation) to evaluate changes in maternal vascular function throughout pregnancy.

All measurements were performed by the same trained investigator according to the manufacturer’s recommendations and the expert consensus document on arterial stiffness assessment [4]. Before each examination, participants rested in the supine position for 10 min in a quiet, temperature-controlled room. To minimize external influences on vascular function, participants were instructed to refrain from smoking and consuming caffeine-containing food or beverages for at least 3 h before the examination and from alcohol consumption for at least 10 h. Participants remained awake, refrained from speaking, and avoided unnecessary movement throughout the assessment. Whenever necessary, repeated measurements were performed to ensure adequate recording quality, and only recordings fulfilling the manufacturer’s quality-control criteria were included in the final analysis.

Pulse wave velocity (PWVao) was considered the primary indicator of arterial stiffness, whereas the augmentation index (AIx) served as a complementary measure of arterial wave reflection and vascular compliance. Given the physiological cardiovascular adaptations that occur during pregnancy, arterial stiffness parameters were interpreted within the study population rather than according to reference values established for non-pregnant adults.

### 2.5. Laboratory Assessment

Venous blood samples were collected from all participants during the same study visit in which arterial stiffness measurements were performed. Blood samples were obtained by standard venipuncture into gel-separator tubes containing a clot activator, allowed to clot at room temperature, and centrifuged for serum separation according to standardized laboratory procedures. Serum aliquots were transferred into sterile polypropylene tubes and stored at −20 °C until biochemical analysis.

Routine laboratory investigations included total cholesterol, high-density lipoprotein cholesterol (HDL-C), low-density lipoprotein cholesterol (LDL-C), triglycerides, and 24 h urinary protein excretion. Proteinuria was quantified as milligrams per 24 h as part of the routine diagnostic evaluation of women with preeclampsia.

Serum interleukin-6 (IL-6) concentrations were measured using a commercially available enzyme-linked immunosorbent assay (ELISA) kit (BT LAB, Biosystems, Shanghai, China) according to the manufacturer’s instructions. Serum samples were incubated in microplate wells pre-coated with anti-human IL-6 antibodies, followed by the addition of a biotinylated detection antibody and streptavidin–horseradish peroxidase. After a 60 min incubation, unbound conjugate was removed through five automated washing cycles using a ELISA Reader and Washer (Analytical Technologies Ltd., Vadodara, India, model AT2960R). A chromogenic substrate solution was subsequently added, producing a colorimetric reaction proportional to the IL-6 concentration. The reaction was terminated with an acidic stop solution, and optical density was measured at 450 nm using an ELISA microplate reader. Serum IL-6 concentrations were calculated from a standard calibration curve ranging from 2 to 600 ng/L, with a lower limit of detection of 1.03 ng/L. Serum IL-6 was measured during the second-trimester assessment (20–24 weeks of gestation), concurrently with the arterial stiffness evaluation. IL-6 was not reassessed during the third-trimester vascular assessment.

All laboratory analyses were performed in the accredited Clinical Laboratory of the Emergency Clinical Hospital “Pius Brînzeu”, Timișoara, following standardized operating procedures and internal quality-control protocols.

### 2.6. Statistical Analysis

Statistical analyses were performed using IBM SPSS Statistics v31.0 (IBM Corp., Armonk, NY, USA) and R (R Foundation for Statistical Computing, Vienna, Austria).

Continuous variables were assessed for normality using the Shapiro–Wilk test. Normally distributed data are presented as mean ± standard deviation (SD), whereas non-normally distributed variables are expressed as median and interquartile range (IQR). Categorical variables are presented as frequencies and percentages.

Associations between serum IL-6 concentrations and arterial stiffness parameters were evaluated using Pearson’s or Spearman’s correlation coefficients, depending on data distribution. Multiple linear regression analyses were performed to identify independent predictors of pulse wave velocity, with maternal age, body mass index, systolic blood pressure, proteinuria, and serum IL-6 concentration entered as independent variables. These covariates were selected based on their potential relevance to arterial stiffness and their representation of major demographic, anthropometric, hemodynamic, renal, and inflammatory factors. Additional multivariable regression analyses were conducted to investigate the associations between inflammatory status and neonatal outcomes.

All statistical tests were two-sided, and a *p* value < 0.05 was considered statistically significant.

## 3. Results

### 3.1. Baseline Characteristics of the Study Population

A total of 87 pregnant women diagnosed with preeclampsia were included in the final analysis. The baseline demographic, clinical, hemodynamic, laboratory, and neonatal characteristics are summarized in Table 1.

The mean maternal age was 37.59 ± 2.65 years, and the mean body mass index (BMI) was 30.41 ± 2.70 kg/m^2^, indicating that the majority of participants were overweight or obese. The mean gestational age at assessment was 32.68 ± 3.10 weeks. Forty-one percent of participants were smokers.

Women presented with severe hypertension, with mean systolic and diastolic blood pressures of 195.55 ± 19.16 mmHg and 120.82 ± 12.35 mmHg, respectively. The mean heart rate was 91.77 ± 9.22 beats/min.

Assessment of arterial stiffness demonstrated a progressive increase during pregnancy. Mean aortic pulse wave velocity (PWVao) increased from 10.94 ± 1.36 m/s during the second trimester to 12.46 ± 1.70 m/s during the third trimester. Similarly, the augmentation index (AIx) increased from 1.26 ± 0.85% to 3.66 ± 1.32%, suggesting progressive deterioration of vascular function as pregnancy advanced.

Laboratory analyses showed a mean serum IL-6 concentration of 31.01 ± 35.61 pg/mL, although values varied considerably among participants (range 1.2–145.4 pg/mL). Mean proteinuria was 1.20 ± 0.70 g/24 h. The lipid profile was characterized by elevated total cholesterol (266.34 ± 27.19 mg/dL), LDL cholesterol (192.29 ± 19.67 mg/dL), and triglycerides (334.25 ± 115.38 mg/dL), whereas HDL cholesterol remained relatively low (26.41 ± 3.72 mg/dL). The mean hemoglobin concentration was 10.87 ± 0.97 g/dL.

Regarding neonatal outcomes, the mean birth weight was 2491.72 ± 785.18 g, and the median Apgar score at 1 min was 8 (IQR 6–8). Cesarean section was performed in 75 women (86.2%).

### 3.2. Changes in Arterial Stiffness During Pregnancy

Comparison of vascular parameters between the second and third trimesters demonstrated a clear increase in arterial stiffness during pregnancy.

**Figure 2:**
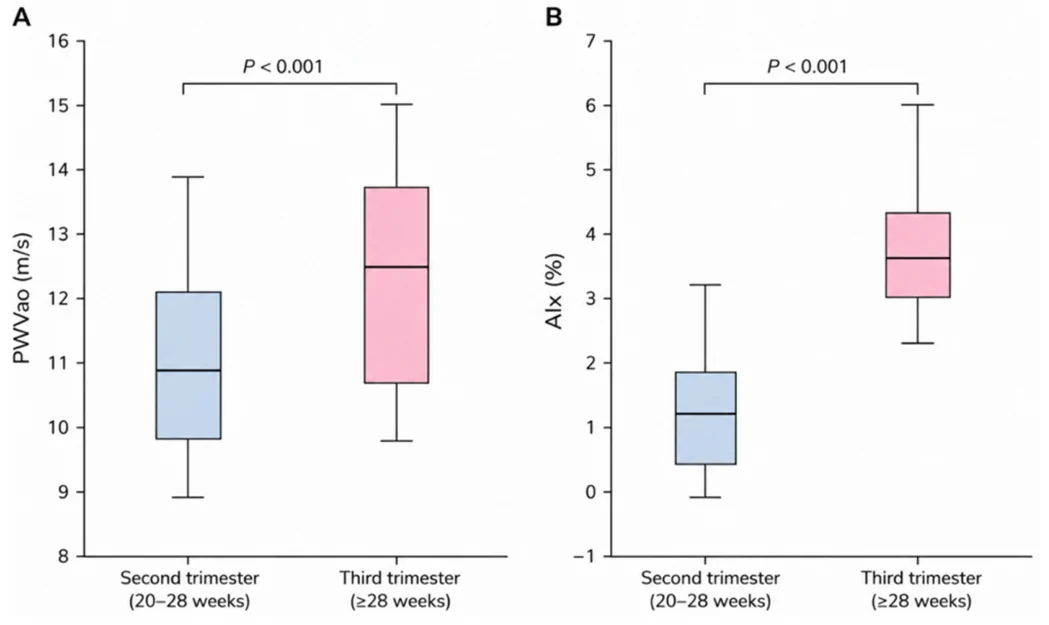
Changes in arterial stiffness parameters between the second and third trimesters in women with preeclampsia. (**A**) Aortic pulse wave velocity (PWVao). (**B**) Augmentation index (AIx). Box plots display the median (horizontal line), interquartile range (box), and minimum–maximum values (whiskers). Mean values are presented below each box plot. *p* values indicate the statistical significance of comparisons between the second and third trimesters.

Mean PWVao increased by approximately 1.5 m/s between the second and third trimesters (10.94 ± 1.36 vs. 12.46 ± 1.70 m/s), while AIx increased from 1.26 ± 0.85% to 3.66 ± 1.32% (Figure 2 and Table 2).

These findings indicate progressive impairment of arterial elasticity as pregnancy advanced in women with preeclampsia.

### 3.3. Association Between Serum IL-6 Concentrations and Arterial Stiffness

**Figure 3:**
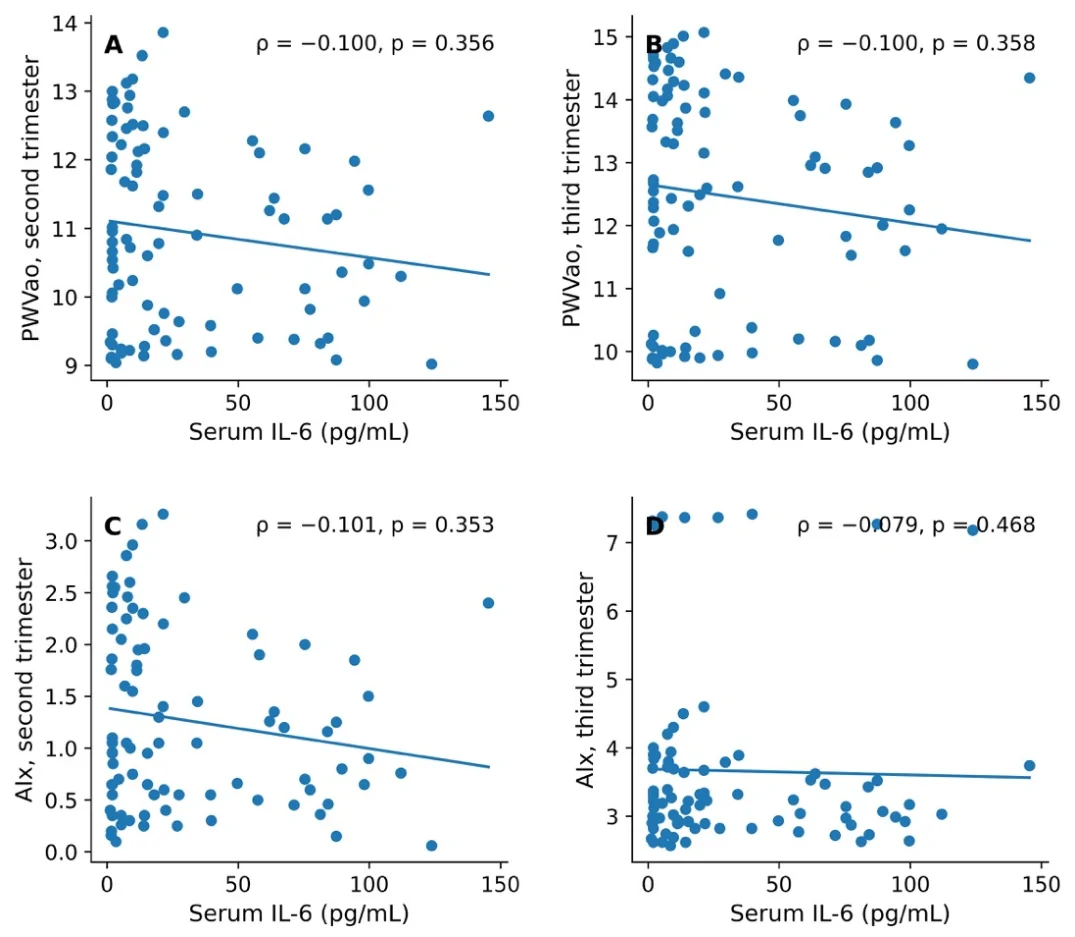
Association between serum interleukin-6 concentrations and arterial stiffness parameters in women with preeclampsia. Scatter plots illustrate the relationships between serum IL-6 concentrations and (**A**) aortic pulse wave velocity (PWVao) during the second trimester, (**B**) PWVao during the third trimester, (**C**) augmentation index (AIx) during the second trimester, and (**D**) AIx during the third trimester. Solid lines represent linear trend lines. No statistically significant correlations were observed between IL-6 concentrations and PWVao or AIx at either assessment (Spearman’s ρ = −0.100, *p* = 0.356; ρ = −0.100, *p* = 0.358; ρ = −0.101, *p* = 0.353; and ρ = −0.079, *p* = 0.468, respectively).

Spearman correlation analysis demonstrated no significant association between serum IL-6 concentrations and arterial stiffness parameters. Specifically, IL-6 was not correlated with PWVao measured during either the second trimester (ρ = −0.100, *p* = 0.356) or the third trimester (ρ = −0.100, *p* = 0.358). Similarly, no significant relationship was observed between IL-6 concentrations and augmentation index during the second trimester (ρ = −0.101, *p* = 0.353) or the third trimester (ρ = −0.079, *p* = 0.468) (Table 3 and Figure 3). These findings suggest that systemic inflammatory activity, reflected by circulating IL-6 concentrations, was not directly associated with arterial stiffness in this cohort of women with preeclampsia.

### 3.4. Association Between Serum IL-6 Concentrations and Clinical Characteristics

**Figure 4:**
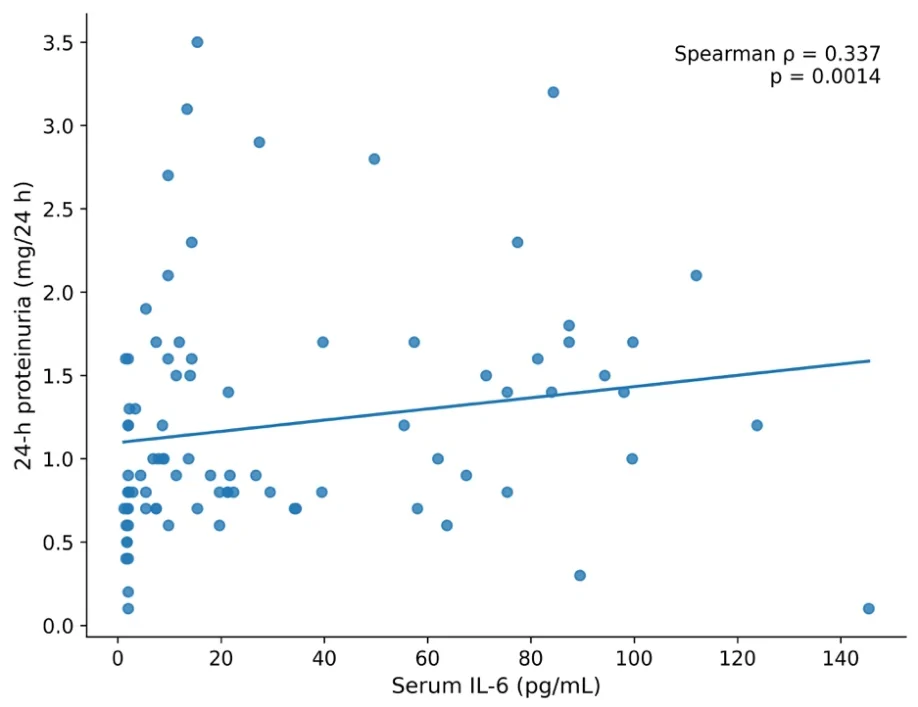
Association between serum interleukin-6 concentrations and 24 h proteinuria in women with preeclampsia.

A significant positive correlation was observed between serum IL-6 concentrations and 24 h proteinuria (ρ = 0.337, *p* = 0.0014), indicating that higher circulating IL-6 levels were associated with greater urinary protein excretion (Figure 4). In contrast, no significant associations were identified between IL-6 concentrations and systolic blood pressure (ρ = −0.113, *p* = 0.299), diastolic blood pressure (ρ = −0.106, *p* = 0.328), maternal body mass index (ρ = −0.094, *p* = 0.385), or maternal age (ρ = 0.053, *p* = 0.624) (Table 4).

### 3.5. Association Between Proteinuria and Arterial Stiffness

Spearman correlation analysis demonstrated no statistically significant associations between 24 h proteinuria and arterial stiffness parameters. Proteinuria was not significantly correlated with PWVao measured during the second trimester (ρ = −0.127, *p* = 0.240) or the third trimester (ρ = −0.118, *p* = 0.277). Similarly, no significant associations were observed between proteinuria and AIx during the second trimester (ρ = −0.127, *p* = 0.242) or the third trimester (ρ = −0.129, *p* = 0.235). These findings indicate that, despite the significant association between IL-6 and proteinuria, proteinuria was not directly associated with the mechanical indices of arterial stiffness in this cohort (Table 5). 

### 3.6. Associations Between Inflammatory and Vascular Parameters and Neonatal Outcomes

Correlation analyses were performed to determine whether systemic inflammation, proteinuria, and maternal arterial stiffness were associated with neonatal outcomes. No statistically significant associations were observed between serum IL-6 concentrations and birth weight (ρ = 0.046, *p* = 0.674) or 1 min Apgar score (ρ = 0.109, *p* = 0.314). Similarly, proteinuria was not significantly correlated with birth weight (ρ = −0.001, *p* = 0.996) or Apgar score (ρ = −0.028, *p* = 0.800). Third-trimester PWVao was also not significantly associated with birth weight (ρ = −0.131, *p* = 0.227) or Apgar score (ρ = −0.137, *p* = 0.206) (Table 6).

### 3.7. Multivariable Predictors of Arterial Stiffness

**Figure 5:**
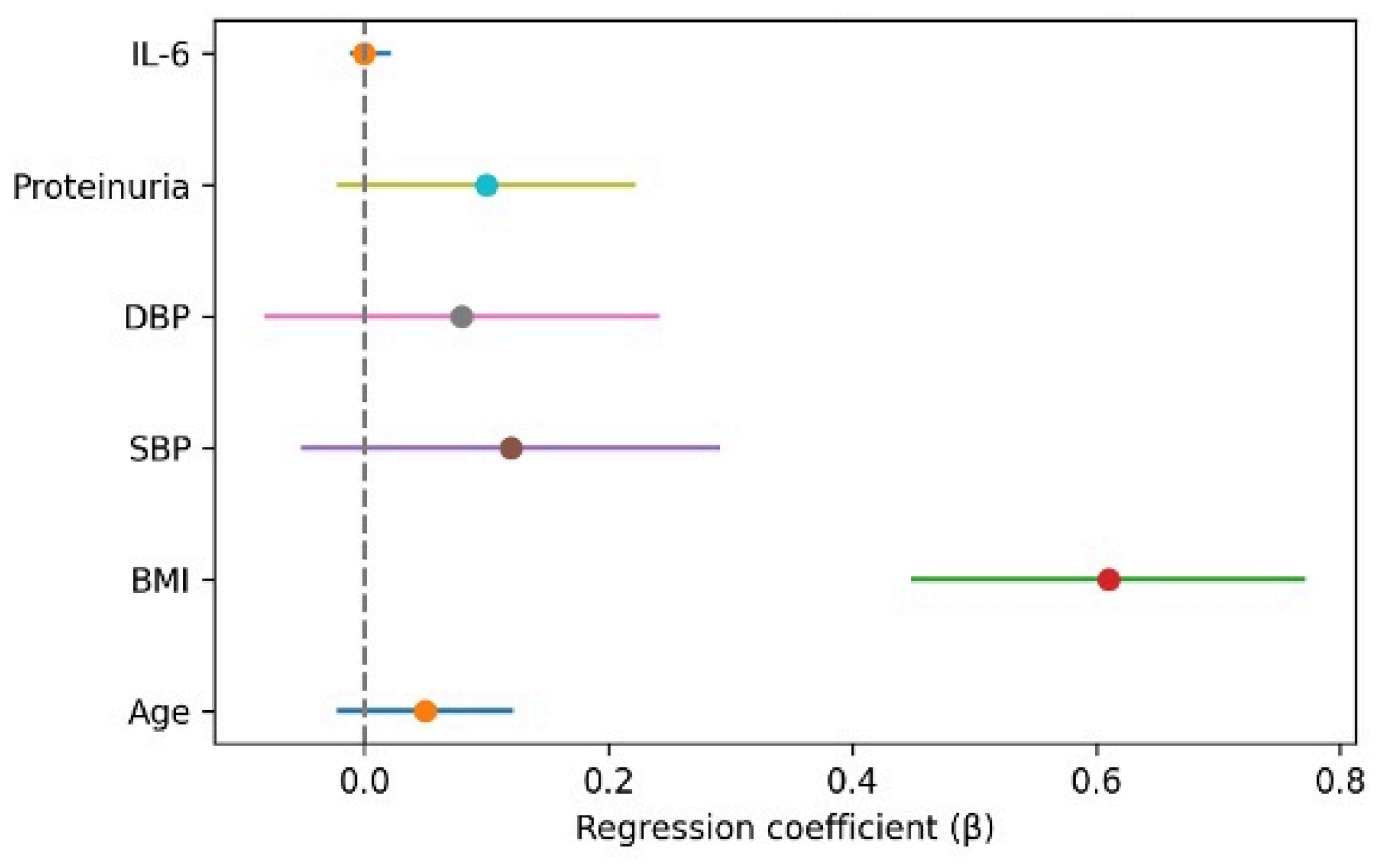
Forest plot of independent predictors of third-trimester pulse wave velocity (PWVao) in women with preeclampsia. The plot displays regression coefficients (β) and 95% confidence intervals for maternal age, body mass index (BMI), systolic blood pressure (SBP), diastolic blood pressure (DBP), proteinuria, and serum interleukin-6 (IL-6). The dashed vertical line represents the null value (β = 0).

A multivariable linear regression model was constructed to identify independent predictors of third-trimester PWVao. Maternal age, BMI, systolic blood pressure, proteinuria, and serum IL-6 concentration were entered as independent variables. The model explained 83.8% of the variance in third-trimester PWVao (R^2^ = 0.838; adjusted R^2^ = 0.828; *p* < 0.001). BMI was the only significant independent predictor of third-trimester PWVao (β = 0.634, 95% CI: 0.469–0.799, *p* < 0.001). Maternal age (β = −0.0038, *p* = 0.897), systolic blood pressure (β = −0.0083, *p* = 0.478), proteinuria (β = −0.0049, *p* = 0.965), and serum IL-6 (β = 0.0009, *p* = 0.694) were not independently associated with third-trimester PWVao (Table 7 and Figure 5). These findings indicate that maternal adiposity may contribute more substantially to arterial stiffness than systemic inflammatory activity in women with preeclampsia.

## 4. Discussions

Preeclampsia is a multisystem disorder involving abnormal placentation, maternal endothelial dysfunction, systemic inflammation, altered vascular reactivity, and progressive cardiovascular maladaptation. In this study, we examined the relationships among systemic inflammation, measured by serum interleukin-6; renal involvement, assessed through 24 h proteinuria; and maternal arterial stiffness, evaluated using PWVao and AIx. Arterial stiffness increased between the second and third trimesters. Serum IL-6 showed a significant positive correlation with proteinuria, whereas its correlations with PWVao and AIx were not significant. Proteinuria also showed no significant association with either arterial stiffness parameter. In the multivariable analysis, BMI was the only independent predictor of third-trimester PWVao. Taken together, these results suggest that systemic inflammation, renal endothelial injury, and mechanical arterial stiffening are connected features of preeclampsia, although they may not arise through the same biological pathway.

### 4.1. Progressive Increase in Arterial Stiffness During Pregnancy

A main finding of this study was the progressive rise in arterial stiffness during pregnancy. Mean PWVao rose by approximately 1.5 m/s, from 10.94 ± 1.36 m/s in the second trimester to 12.46 ± 1.70 m/s in the third. Over the same period, AIx increased from 1.26 ± 0.85% to 3.66 ± 1.32%. Together, these changes in PWVao and AIx point to worsening large-artery elasticity and altered arterial wave reflection as pregnancy progressed in women with preeclampsia.

These findings align with broader research showing greater arterial stiffness in preeclampsia. In a recent systematic review and meta-analysis of 21 studies involving 6488 participants, PWVao was significantly higher among women with preeclampsia than among those without pregnancy-associated disease. The pooled mean difference was 0.67 m/s (95% CI, 0.51–0.83; *p* < 0.00001). The authors concluded that PWVao may be a useful marker of vascular dysfunction during pregnancy [20].

Our findings align with earlier prospective research on the predictive value of PWV. Katsipi et al. found that second-trimester PWVao was approximately 30% higher in women who later developed preeclampsia than in normotensive controls [21]. Its predictive performance was especially strong for early-onset preeclampsia, and combining PWVao with sFlt-1 improved prediction further. In a similar study, Ivan et al. showed that PWVao could help identify women at high risk of preeclampsia and reported positive associations between PWVao and proteinuria [22].

The present study builds on these observations, showing that PWVao and AIx increase during pregnancy among women with established preeclampsia. Arterial stiffness, then, may not merely reflect a pre-existing cardiovascular characteristic; it may also indicate the progressive vascular burden associated with preeclampsia.

The rise in AIx adds information that PWVao alone does not provide. PWVao mainly captures the stiffness of large elastic arteries, while AIx is affected by wave reflection within the arteries and by characteristics of the peripheral vasculature. Taken together, the increases in both measures suggest that vascular dysfunction in preeclampsia includes reduced arterial elasticity as well as changes in wave reflection [23].

Recent evidence suggests that vascular abnormalities linked to hypertensive disorders of pregnancy can persist after delivery. In a 2025 systematic review and meta-analysis of 856 women, AIx and carotid-femoral PWVao remained significantly elevated more than six weeks postpartum among those with a history of hypertensive disorders of pregnancy [24]. This supports the concept that the vascular phenotype observed during preeclampsia may have implications extending beyond the pregnancy itself.

### 4.2. IL-6 and Arterial Stiffness

Although inflammation is well established as a feature of preeclampsia, this study found no significant association between serum IL-6 and arterial stiffness. During the second trimester, IL-6 was not significantly correlated with PWVao (ρ = −0.100, *p* = 0.356), and the same was true during the third trimester (ρ = −0.100, *p* = 0.358). IL-6 also showed no significant association with AIx in either trimester. This finding matters because, in established preeclampsia, circulating IL-6 concentration alone may not directly capture the mechanical properties of the arterial wall. Still, the lack of an IL-6–PWVao association does not mean that inflammation is unimportant in preeclampsia. It may instead indicate that inflammatory activation and arterial stiffening are, at least in part, independent processes.

Recent evidence points strongly to systemic inflammation as a factor in preeclampsia. In a 2024 systematic review and meta-analysis of 25 studies, maternal concentrations of several inflammatory markers—including high-sensitivity CRP and IL-6—were higher among women with preeclampsia [25]. The results support the biological relevance of IL-6, while also suggesting that inflammatory biomarkers can capture different parts of the disease’s pathophysiology.

Preeclampsia involves a complex inflammatory response, with numerous cytokines, chemokines, acute-phase proteins, oxidative pathways, and damage-associated molecular patterns. Recent work has drawn particular attention to S100A8 and S100A9, pointing to their possible importance in innate immune activation and inflammatory signaling at the maternal–placental interface [26]. Against that background, the absence of a correlation between IL-6 and PWVao in the present study may suggest that no single inflammatory biomarker is sufficient to account for the mechanical vascular phenotype.

IL-6 may be more closely tied to endothelial activation and injury in specific organs than to established remodeling of the arterial wall. PWVao captures the combined influence of vascular structure, extracellular matrix composition, smooth-muscle tone, blood-pressure loading, and metabolic factors. A circulating cytokine can therefore remain biologically relevant without showing an independent association with PWVao once these other determinants are taken into account.

### 4.3. IL-6 and Proteinuria: A Link Between Inflammation and Renal Involvement

Unlike the lack of an association between IL-6 and arterial stiffness, this study found a significant positive correlation between serum IL-6 and 24 h proteinuria (ρ = 0.337, *p* = 0.0014). The finding suggests that, in this cohort, systemic inflammatory activation may be more closely linked to renal endothelial involvement than to mechanical arterial stiffness.

Proteinuria is an important clinical sign of glomerular endothelial dysfunction in preeclampsia. Because the glomerular endothelium is especially sensitive to the circulating antiangiogenic, inflammatory, and endothelial factors linked to preeclampsia [27], the positive association between IL-6 and proteinuria suggests that greater systemic inflammatory activity corresponds to more marked renal involvement.

This interpretation is further supported by the broader cardio-renal framework described by Twardawa et al. [28], who emphasized the close relationship between vascular alterations and circulating biomarkers across different stages of chronic kidney disease. Their findings suggest that renal dysfunction and vascular abnormalities may represent interconnected components of systemic endothelial and cardiovascular injury rather than isolated organ-specific processes. Within the context of preeclampsia, the association observed between IL-6 and proteinuria may similarly reflect a shared systemic inflammatory and endothelial pathway affecting the renal microvasculature. This relationship may occur independently of changes in large-artery stiffness, which could explain the absence of a significant direct association between proteinuria and arterial stiffness parameters in our cohort.

Petre et al.’s findings are relevant here. Their study focused specifically on CRP and IL-6 in obesity-associated preeclampsia, highlighting the relationship among obesity, inflammatory activation, and preeclampsia [29]. The study design and population were different from ours, but the results support the view that IL-6 may offer information about the inflammatory phenotype of preeclampsia.

Interestingly, no significant correlation was found between proteinuria and either PWVao or AIx in the present cohort. That finding differs from the report by Ivan et al., where proteinuria was positively associated with PWVao among women at high risk of preeclampsia [22]. Differences in the study populations and disease stage may account for the discrepancy. Ivan et al. examined women at high risk of developing preeclampsia, while this study included women with established preeclampsia. The relationship between proteinuria and vascular stiffness may also be affected by disease severity and duration, antihypertensive treatment, metabolic characteristics, and the timing of measurements.

The findings suggest that renal endothelial injury and mechanical arterial stiffening may follow different trajectories rather than progressing together. Proteinuria may reflect mainly organ-specific endothelial dysfunction, while PWVao captures the accumulated mechanical effects of vascular remodeling and hemodynamic stress.

The absence of a significant association between IL-6 and PWVao or AIx may also reflect the complex and multifactorial nature of vascular dysfunction in preeclampsia. Arterial stiffness is influenced not only by inflammatory signaling but also by blood pressure loading, vascular remodeling, extracellular matrix alterations, vascular smooth muscle tone, and metabolic factors. In this context, IL-6 may reflect the systemic inflammatory burden of preeclampsia without being a direct determinant of large-artery mechanical properties. This interpretation is supported by our multivariable analysis, in which BMI, rather than IL-6, was the only independent predictor of third-trimester PWVao. Thus, the lack of an association between IL-6 and arterial stiffness should not be interpreted as evidence against a role of inflammation in preeclampsia, but rather as an indication that circulating IL-6 alone may not adequately capture the complex mechanisms underlying large-artery stiffening [15].

### 4.4. Maternal Adiposity as the Principal Independent Determinant of PWV

The multivariable analysis yielded a particularly important result: BMI was the sole independent predictor of third-trimester PWVao (β = 0.6340; 95% CI, 0.469–0.799; *p* < 0.001). No independent association with third-trimester PWVao was observed for maternal age, SBP, proteinuria, or serum IL-6. Clinically, this result matters because the cohort’s mean BMI was approximately 30 kg/m^2^, reflecting a predominantly overweight/obese population. BMI’s strong relationship with arterial stiffness suggests that metabolic factors may exert a more direct effect on the mechanical vascular phenotype than circulating IL-6 in women with established preeclampsia.

The relatively high blood pressure values observed in our cohort are consistent with the clinical characteristics of women with established preeclampsia and reflect the hypertensive phenotype of the study population. Similarly, the relatively high BMI values indicate a cohort with increased maternal adiposity. The independent association between BMI and third-trimester PWVao suggests that maternal adiposity may contribute to vascular stiffening in preeclampsia. However, BMI measured during pregnancy should be interpreted cautiously, as it may reflect not only adiposity but also gestational age, fetal and placental growth, physiological fluid expansion, and, in preeclampsia, fluid retention and edema. Therefore, the observed association between BMI and PWVao may not exclusively reflect baseline maternal adiposity.

The connection between obesity, inflammation, and preeclampsia has been well established. Because adipose tissue is metabolically active, it contributes to persistent low-grade inflammation by releasing cytokines and adipokines. Petre et al. reported an association among obesity, IL-6, CRP, and preeclampsia [29]. Our findings, however, indicate that obesity’s effects on the vasculature extend beyond inflammation.

Several processes could account for the independent relationship between BMI and PWV: heightened sympathetic activity, oxidative stress, reduced nitric oxide bioavailability, endothelial dysfunction, insulin resistance, changes in vascular smooth-muscle function, and remodeling of the extracellular matrix [30]. Together, they may increase arterial stiffness without depending on circulating IL-6.

Recent intervention research underscores the clinical importance of modifiable vascular determinants. In a study by Petre et al., a structured, personalized exercise program affected hemodynamic parameters and arterial stiffness among pregnant women [31]. Alongside the strong BMI–PWVao relationship found in our cohort, these results point to the need for further study of lifestyle and metabolic interventions that could reduce maternal vascular dysfunction.

### 4.5. Arterial Stiffness as a Multifactorial Component of Preeclampsia

The results point to arterial stiffness in preeclampsia arising from several contributing factors. IL-6 was significantly associated with proteinuria, but neither marker showed a significant relationship with PWVao or AIx. BMI, by contrast, was the strongest independent determinant of PWVao. This distinction matters when considering what PWVao means biologically. It should not be treated as a direct surrogate for systemic inflammation. Instead, PWVao represents the arterial system’s combined mechanical response to several influences, including blood pressure, vascular smooth-muscle tone, structural remodeling, metabolic status, oxidative stress, and endothelial dysfunction [32].

Recent studies have pointed to other potentially modifiable factors that may affect arterial stiffness during pregnancy. In pregnant women with preeclampsia and pregnancy-induced hypertension, Iurciuc et al. found an association between vitamin D deficiency and arterial stiffness, with possible implications for fetal development [3]. These findings support the view that, alongside inflammation, metabolic and nutritional factors also influence vascular stiffness in preeclampsia.

Xu et al.’s 2024 meta-analysis adds further evidence that PWVao is a marker of vascular dysfunction in pregnancy-associated disease [20]. Across 21 studies, PWVao was significantly higher in preeclampsia and in other pregnancy-associated metabolic and hypertensive conditions.

Consequently, a multidimensional approach combining PWVao with BMI, proteinuria, inflammatory biomarkers, angiogenic factors, and other cardiovascular measures may be more informative than any individual biomarker.

### 4.6. Relationship with the Broader Cardiovascular Phenotype of Hypertensive Pregnancy

The greater arterial stiffness found in our study should be viewed alongside the wider cardiovascular features of hypertensive pregnancy. Preeclampsia affects more than the arterial system; it also alters cardiac structure, ventricular function, and autonomic regulation.

Bogdan et al. recently reported abnormalities in heart-rate variability, global longitudinal strain, and diastolic function among pregnant women with hypertensive disorders, with the most pronounced findings seen in those with severe preeclampsia [33]. Their results support the present study, indicating that maternal cardiovascular maladaptation in preeclampsia affects several interconnected domains.

Accordingly, PWVao and AIx are better viewed as elements of a broader cardiovascular phenotype than as standalone markers. Future studies that examine arterial stiffness alongside central blood pressure, heart-rate variability, echocardiographic strain, and biochemical markers may offer a fuller assessment of maternal cardiovascular risk.

### 4.7. Lack of Association Between Inflammatory and Vascular Markers and Neonatal Outcomes

The present study found no significant associations of IL-6, proteinuria, PWVao, or AIx with neonatal outcomes. Specifically, IL-6 was not significantly correlated with birth weight (ρ = 0.046, *p* = 0.674) or the 1 min Apgar score (ρ = 0.109, *p* = 0.314). Proteinuria likewise showed no significant association with either birth weight or Apgar score. Third-trimester PWVao also showed no significant relationship with birth weight (ρ = −0.131, *p* = 0.227) or Apgar score (ρ = −0.137, *p* = 0.206). These findings suggest that, within this cohort, the inflammatory and vascular measures assessed in the study did not account for short-term neonatal condition. Still, the negative results warrant caution. Birth weight and the 1 min Apgar score are broad clinical endpoints; they may not capture placental insufficiency, fetal growth restriction, or fetal cardiovascular adaptation in full.

Recent evidence indicates that adverse maternal and perinatal outcomes linked to preeclampsia arise from multiple factors. Prediction improves when clinical, biochemical, and obstetric variables are considered together rather than when assessment depends on a single biomarker [34]. Thus, the absence of significant correlations in our cohort does not rule out clinically relevant associations with specific outcomes, including fetal growth restriction, small-for-gestational-age birth, preterm delivery, or neonatal intensive-care admission.

### 4.8. Clinical Implications

These findings may have several clinical implications. The rise in PWVao and AIx from the second to the third trimester supports repeated assessment of maternal vascular function in women with preeclampsia, since one measurement may not fully capture the changing course of vascular dysfunction. IL-6 and PWVao seem to offer complementary information rather than duplicate information. IL-6 was linked to proteinuria, pointing to possible connections with systemic inflammation and renal endothelial involvement. PWV, by contrast, was mainly associated with maternal adiposity. That distinction could be useful in developing multidimensional approaches to risk stratification.

Third, BMI should be taken into account when arterial stiffness is interpreted in women with preeclampsia. Its strong independent association with third-trimester PWVao suggests that metabolic risk may make a substantial contribution to maternal vascular dysfunction.

Previous research also indicates that PWVao may help predict preeclampsia, particularly when used alongside angiogenic markers such as sFlt-1 [21]. Future models could potentially integrate PWVao with BMI, proteinuria, IL-6, angiogenic biomarkers, and other maternal cardiovascular characteristics.

### 4.9. Strengths and Limitations

The study’s main strength lies in examining systemic inflammation, renal involvement, and arterial stiffness together in the same group of women with preeclampsia. Repeated measurements of PWVao and AIx in the second and third trimesters also show how maternal vascular dysfunction changes over time, rather than capturing it at only one point. Another strength was the standardized, non-invasive assessment of arterial stiffness with the Arteriograph system, carried out under controlled measurement conditions. The study also used correlation analyses alongside multivariable regression, making it possible to assess the independent contributions of BMI, blood pressure, proteinuria, and IL-6 to third-trimester PWVao.

Several limitations should nevertheless be acknowledged. The study included only 87 participants, which limited statistical power to detect modest associations. Its single-center design, conducted at a tertiary referral center, may also restrict generalizability. In addition, because the cohort comprised exclusively women with preeclampsia, the study could not establish whether the observed vascular and inflammatory relationships differ from those in normotensive pregnancies or among women at risk of preeclampsia.

An additional limitation of this study is the absence of a normotensive pregnant control group. As the study population consisted exclusively of women with preeclampsia, it was not possible to determine whether the observed alterations in PWVao and AIx were specifically related to preeclampsia or partly reflected physiological cardiovascular adaptations occurring during pregnancy. Likewise, IL-6 levels and their association with proteinuria could not be evaluated against values observed in uncomplicated pregnancies. Accordingly, our findings primarily characterize the interplay between inflammatory, renal, and vascular parameters within a preeclamptic population and should not be interpreted as evidence of disease-specific differences compared with normal pregnancy. Future studies incorporating appropriately matched normotensive pregnant controls are needed to better distinguish preeclampsia-related vascular and inflammatory abnormalities from physiological gestational changes.

Gestational age represents another potential confounding factor that should be considered when interpreting our findings. Inflammatory activity, proteinuria, and vascular properties may vary throughout pregnancy, while the clinical expression of preeclampsia may also change with disease progression. Although arterial stiffness was evaluated within predefined gestational intervals, gestational age was not included as an adjustment variable in the correlation or multivariable analyses. Consequently, a potential influence of advancing gestation on the observed association between IL-6 and 24 h proteinuria cannot be entirely excluded. This relationship should therefore be interpreted as an association within the studied preeclamptic population rather than as evidence of an independent effect of inflammation on renal involvement. Longitudinal studies incorporating gestational age and disease severity into adjusted models are needed to better define the temporal interplay between systemic inflammation and renal manifestations of preeclampsia.

Fourth, IL-6 captures only part of the inflammatory response. Thus, the absence of an association between IL-6 and PWVao does not show that inflammation overall is unrelated to arterial stiffness. Future research should examine wider inflammatory profiles, including CRP, TNF-α, other cytokines, and emerging markers such as S100A8/A9 [25,26]. Residual confounding also remains possible, including confounding related to disease severity, treatment, nutritional status, metabolic characteristics, placental factors, and other unmeasured variables.

The interpretation of BMI as an independent predictor of third-trimester PWVao should also be considered with caution. BMI was assessed during both the second and third trimesters and therefore reflects maternal body weight during pregnancy rather than pre-pregnancy adiposity alone. Maternal weight during pregnancy may be influenced by gestational age, fetal and placental growth, physiological plasma volume expansion, and, in preeclampsia, fluid retention and edema. Consequently, the observed association between BMI and third-trimester PWVao may partly reflect pregnancy-related changes in body weight rather than baseline metabolic status alone. Furthermore, because gestational age was not included as a covariate in the multivariable model, a potential confounding effect of advancing gestation cannot be completely excluded.

In addition, IL-6 was assessed as a single circulating inflammatory biomarker and only at the second-trimester assessment. Although IL-6 is an important mediator of systemic inflammation, it represents only one component of the complex inflammatory and endothelial pathways involved in preeclampsia. A single measurement may therefore not fully capture temporal changes in inflammatory activity or its potential relationship with the progression of vascular dysfunction.

### 4.10. Future Research

Future research should draw on larger, multicenter prospective cohorts that include normotensive pregnant women, women with gestational hypertension, and patients representing different preeclampsia phenotypes and stages. Repeated measurements of PWVao and AIx should be paired with inflammatory, angiogenic, metabolic, and cardiac biomarkers.

A key question is whether combining BMI, PWVao, proteinuria, IL-6, and angiogenic biomarkers predicts severe maternal and fetal complications more accurately than conventional clinical parameters alone.

The fact that arterial stiffness may be modifiable deserves closer study. Findings on structured physical activity [31,35,36] and vitamin D status [3,37] indicate that some factors contributing to maternal vascular dysfunction could respond to intervention. Prospective studies should examine whether reducing maternal adiposity or correcting particular metabolic and nutritional abnormalities can slow the progression of PWVao during pregnancy.

Long-term follow-up matters as well. In a recent meta-analysis, Mbongozi et al. found that increased arterial stiffness may persist beyond six weeks postpartum in women with previous hypertensive disorders of pregnancy [24]. Longitudinal studies linking antenatal PWVao trajectories to postpartum vascular function and later cardiovascular events could clarify whether the increase in PWVao observed during preeclampsia represents a transient pregnancy-related phenomenon or an early manifestation of long-term cardiovascular risk.

## 5. Conclusions

Preeclampsia involves worsening maternal vascular dysfunction, with PWVao and AIx increasing from the second to the third trimester. Although serum IL-6 was significantly associated with proteinuria, pointing to a possible link between systemic inflammation and renal endothelial involvement, it showed no direct association with arterial stiffness. By the third trimester, BMI was the only independent predictor of PWVao, underscoring the contribution of maternal adiposity to vascular dysfunction.

These findings indicate that inflammation, proteinuria, and arterial stiffness are separate yet related features of preeclampsia. Assessing inflammatory, renal, metabolic, and vascular measures together may provide a clearer picture of maternal cardiovascular health and improve risk stratification.

Future studies should draw on larger, multicenter cohorts and follow participants longitudinally after delivery, while also examining broader panels of inflammatory and angiogenic biomarkers. They should also assess whether combining BMI, PWVao, IL-6, proteinuria, and angiogenic markers improves the prediction of severe maternal and neonatal outcomes and helps identify women at increased long-term cardiovascular risk.

## Figures and Tables

**Table 1:** Baseline demographic, clinical, hemodynamic, laboratory, and neonatal characteristics of the study population.

Variable	Mean ± SD	Median (IQR)	Range
**Demographic characteristics**			
Maternal age (years)	37.59 ± 2.65	38 (37–39)	23–42
Height (cm)	166.84 ± 8.09	167 (160.5–170.0)	153–183
Weight (kg)	84.55 ± 8.52	85 (78–91)	69–100
Body mass index (kg/m^2^)	30.41 ± 2.70	30.09 (28.03–33.10)	27.13–35.11
**Clinical characteristics**			
Systolic blood pressure (mmHg)	195.55 ± 19.16	190 (178–212.5)	173–238
Diastolic blood pressure (mmHg)	120.82 ± 12.35	116 (111–133)	101–141
Heart rate (beats/min)	91.77 ± 9.22	92 (85–96)	76–120
Gestational age (weeks)	32.68 ± 3.10	34 (31–35)	25–37
**Hemodynamic parameters**			
PWVao, second trimester (m/s)	10.94 ± 1.36	10.84 (9.61–12.14)	9.02–13.86
PWVao, third trimester (m/s)	12.46 ± 1.70	12.62 (10.65–13.99)	9.80–15.07
AIx, second trimester (%)	1.26 ± 0.85	1.05 (0.55–1.95)	0.06–3.26
AIx, third trimester (%)	3.66 ± 1.32	3.23 (2.92–3.73)	2.57–7.42
**Laboratory parameters**			
Hemoglobin (g/dL)	10.87 ± 0.97	10.8 (10.15–11.60)	8.8–12.9
Total cholesterol (mg/dL)	266.34 ± 27.19	268 (246–287)	199–324
HDL cholesterol (mg/dL)	26.41 ± 3.72	26 (24–28)	22–39
LDL cholesterol (mg/dL)	192.29 ± 19.67	197 (176.5–209.5)	152–220
Triglycerides (mg/dL)	334.25 ± 115.38	354 (225–430.5)	153–570
Serum IL-6 (pg/mL)	31.01 ± 35.61	14.0 (3.9–56.4)	1.2–145.4
Proteinuria (g/24 h)	1.20 ± 0.70	1.0 (0.7–1.6)	0.1–3.5
**Neonatal characteristics**			
Birth weight (g)	2491.72 ± 785.18	2630 (2255–3085)	750–3550
Apgar score at 1 min	7.03 ± 1.94	8 (6–8)	0–9
Categorical variables	n (%)		
Smoking	36 (41.4)	–	–
Male neonates	54 (62.1)	–	–
Female neonates	33 (37.9)	–	–
Cesarean delivery	75 (86.2)	–	–

Abbreviations: AIx, augmentation index; BMI, body mass index; HDL, high-density lipoprotein; IL-6, interleukin-6; LDL, low-density lipoprotein; PWVao, aortic pulse wave velocity; SD, standard deviation; IQR, interquartile range.

**Table 2:** Comparison of arterial stiffness parameters between the second and third trimesters.

Parameter	Second Trimester	Third Trimester
PWVao (m/s)	10.94 ± 1.36	12.46 ± 1.70
AIx (%)	1.26 ± 0.85	3.66 ± 1.32

**Table 3:** Correlation between serum IL-6 concentrations and arterial stiffness parameters.

Variable	Spearman’s ρ	*p*-Value
PWVao (second trimester)	−0.100	0.356
PWVao (third trimester)	−0.100	0.358
AIx (second trimester)	−0.101	0.353
AIx (third trimester)	−0.079	0.468

**Table 4:** Correlation between serum IL-6 concentrations and clinical characteristics.

Variable	Spearman’s ρ	*p*-Value
Proteinuria	0.337	0.0014
Systolic blood pressure	−0.113	0.299
Diastolic blood pressure	−0.106	0.328
Body mass index	−0.094	0.385
Maternal age	0.053	0.624

**Table 5:** Correlation between 24 h proteinuria and arterial stiffness parameters.

Variable	Spearman’s ρ	*p*-Value
PWVao, second trimester	−0.127	0.240
PWVao, third trimester	−0.118	0.277
AIx, second trimester	−0.127	0.242
AIx, third trimester	−0.129	0.235

**Table 6:** Correlations between inflammatory/vascular parameters and neonatal outcomes.

Variable	Birth Weight, Spearman’s ρ	*p*-Value	Apgar Score, Spearman’s ρ	*p*-Value
Serum IL-6	0.046	0.674	0.109	0.314
Proteinuria	−0.001	0.996	−0.028	0.800
PWVao, third trimester	−0.131	0.227	−0.137	0.206

**Table 7:** Multivariable linear regression analysis for predictors of third-trimester PWVao.

Predictor	β Coefficient	95% CI	*p*-Value
Maternal age	−0.0038	−0.0624 to 0.0548	0.897
Body mass index	0.6340	0.4690 to 0.7990	<0.001
Systolic blood pressure	−0.0083	−0.0317 to 0.0150	0.478
Proteinuria	−0.0049	−0.2273 to 0.2176	0.965
Serum IL-6	0.0009	−0.0035 to 0.0053	0.694

## Data Availability

The original contributions presented in this study are included in the article. For further inquiries, please contact the corresponding authors.
